# Antifouling Properties of PES Membranes by Blending with ZnO Nanoparticles and NMP–Acetone Mixture as Solvent

**DOI:** 10.3390/membranes8040131

**Published:** 2018-12-14

**Authors:** Abdul Latif Ahmad, Jayasree Sugumaran, Noor Fazliani Shoparwe

**Affiliations:** 1 School of Chemical Engineering, Engineering Campus, Universiti Sains Malaysia, 14300 Nibong Tebal, Malaysia; jeyasree.sugumaran1@gmail.com; 2 Faculty of Bioengineering and Technology, Jeli Campus, Universiti Malaysia Kelantan, 17600 Kelantan, Malaysia; fazliani.s@umk.edu.my

**Keywords:** polyethersulfone, zinc oxide, mixed matrix membrane, humic acid removal, mixed solvent, antifouling

## Abstract

In this study, the antifouling properties of polyethersulfone (PES) membranes blended with different amounts of ZnO nanoparticles and a fixed ratio of N-methyl-2-pyrrolidone (NMP)-acetone mixture as a solvent were investigated. The properties and performance of the fabricated membranes were examined in terms of hydrophilicity, porosity, pore size, surface and cross-section image using scanning electron microscopy (SEM), surface roughness using atomic force microscopy (AFM), pure water flux, and humic acid filtration. Addition of ZnO as expected was found to improve the hydrophilicity as well as to encourage pore formation. However, the agglomeration of ZnO at a higher concentration cannot be avoided even when dissolved in a mixed solvent. The presence of highly volatile acetone contributed to the tight skin layer of the membrane which shows remarkable antifouling ability with the highest flux recovery ratio and negligible irreversible fouling. ZnO NPs in acetone/NMP mixed solvent shows an improvement in flux and rejection, but, the fouling resistance was moderate compared to the pristine membrane.

## 1. Introduction

The contributions of membranes in many industries such as food processing [1], the petrochemical industry [2], energy applications [3,4], as well as wastewater and water purification technology [5] have been highly successful and are much sought-after. Due to the ever expanding world population, healthy water resources have become a luxury for over 2.1 billion people worldwide who do not have access to safely managed water [6]. In order to have clean and accessible water for all, the United Nations has made ‘Clean Water and Sanitation’ their 6th sustainable development goal which is targeted to be achieved by 2030 [7]. Membrane technology obviously has some major advantages as compared to other technologies such as energy efficiency and reasonable cost [8]. However, membrane fouling has been identified as one of the most difficult problems that restrict the use of membranes in industry from a technical and economical point of view [9,10].

Fouling is a phenomenon that occurs when the colloids, particles, macromolecules, salts, etc. are deposited or adsorbed on the pore walls, inside pores or/and the surface of the membrane. However, nonporous membrane mainly experience an external fouling [11]. Fouling can be reversible or irreversible. The weakly bound foulants, which cause the reversible fouling can be removed by a mere hydraulic cleaning method. During irreversible fouling, the foulants have a strong affinity towards the membrane surface and are strongly attached to it and as such require chemical cleaning which may shorten the longevity of the membrane [12].

Fouling control is an important step for membranes to be competitive compared to other technologies. Many researchers have shown that improving the hydrophilicity of the membrane will reduce the hydrophobic interaction between the foulants in the feed and the membrane surface [13,14,15]. Polyethersulfone (PES) is one of the versatile polymers used for the preparation of membranes with high chemical resistance and high glass transition temperature but hydrophobicity of this material leads to severe fouling in the membrane. The PES membrane can be modified to be more hydrophilic by grafting, coating, and blending methods. Blending has a certain advantage compared to coating and grafting as it enables membrane modification during the fabrication stage, while grafting and coating are post-fabrication modifications. Blending is also considered to be the most facile among these three routes.

Incorporation of inorganic nanoparticles (NPs) exists such as multiwalled carbon nanotube (MWCNT) [16], silicon dioxide (SiO_2_) [17], titanium dioxide (TiO_2_) [18], graphene oxide (GO) [19], and zinc oxide (ZnO) [20,21]. The popularity of TiO_2_ as fouling resistant agent has been confirmed by many researchers [22,23]. However, ZnO can be an excellent alternative to TiO_2_ as an antifouling material. The increase of the surface to volume ratio and inexpensiveness make ZnO a potential candidate that can meet the demand for efficient and lower-cost NPs [10]. Since the size distribution and surface area are not related to toxicity, the use of ZnO-NPs does not increase toxicity [10]. Balta et al. observed an improved hydrophilicity and water permeability upon introduction of ZnO. The adsorption of humic acid (HA) within the membrane structure was also reduced due to ZnO incorporation.

The solvent used to dissolve the polymer can have a significant effect on the morphology of the membrane. Madaeni and Taheri reported significant effects on morphology of polyvinylidene fluoride (PVDF) by varying the solvent used such as NMP, dimethylacetamide (DMAc), and dimethylformamide (DMF). In their study, DMAc was found to result in a highly porous membrane with slightly finger-like macrovoid at the substructure after the skin layer. In the case of DMF, a highly denser membrane with little pores was observed compared to all other solvents [24]. Few researchers have combined two or more solvent mixtures in the casting solution to get a desirable morphology on the membrane [25,26]. Kim et al. showed the possibility to form an integrally-skinned nanofiltration membrane with well-developed skin layer supported by a porous sublayer by manipulating the composition of diethylene glycol dimethyl ether (DGDE) and acetic acid (AA) in NMP solution. This significant change in morphology directly affected the performance of the membrane, with PEG 600 removal maintaining a constant value of up to 83% [25]. Acetone which is a highly volatile solvent used together with low volatile solvent results in membranes with slightly lower permeation coupled with excellent rejection rates as shown by Ahmed et al. [27]. By applying a similar concept, NMP can be used with acetone to create a membrane with similar morphology hence create a similar effect for the performance of the membrane.

To the best of our knowledge, no report has been published regarding the characterization and antifouling properties of fabricated PES/ZnO mixed matrix membranes by using a mixture of acetone–NMP as a solvent. Therefore, in this study, antifouling PES membranes blended with different concentration of ZnO nanoparticles and a fixed ratio of NMP–acetone were fabricated using the phase inversion method. A series of experiments including SEM, AFM, porosity, pore size, and water contact angle measurements were carried out for membrane characterization. The performance and antifouling properties of the fabricated membranes were evaluated by testing the water permeability and humic acid rejection.

## 2. Materials and Methods

### 2.1. Materials

Polyethersulfone (PES, Ultrason E6020P; Mw = 58,000 g/mol) was purchased from BASF (Kuala Lumpur, Malaysia) Acetone was supplied from Merck (Selangor, Malaysia) while N-methyl-2-pyrrolidone (NMP) was purchased from Sigma Aldrich, Malaysia (Selangor, Malaysia). Zinc oxide nanoparticle (40 nm average) and humic acid (HA) were also from Sigma. Deionized water was used throughout the experiment.

### 2.2. Membrane Preparation

In this study, flat sheet membranes were prepared via non-solvent induced phase separation (NIPS). In detail, cast solutions (Table 1) were prepared using the following steps. Mixed solvent, acetone and NMP were mechanically stirred at 400 rpm for 1 h. Then, various amounts of ZnO-NPs (0 wt%, 0.5 wt%, 1.0 wt%, 1.5 wt% and 2 wt%) were dispersed in NMP and acetone solution. The solution was mechanically stirred at 600 rpm for 3 h at room temperature followed by sonication for 1 h. After sonication, the solution was stirred again for another 1 h. PES polymer was dried at 70 °C in a vacuum oven overnight prior to use. The pre-dried PES polymer was slowly added to the dope solution over a period of 2 h to avoid precipitation of the polymer. Then, the solution was stirred at a speed of 500 rpm at a temperature of 60 °C for 24 h followed by degassing. The solution was cast with a casting machine filmograph (K4340 automatic Film Applicator, Elcometer, Manchester, UK) using a casting knife with an opening of 200 µm. The membranes were left on a glass plate at ambient temperature for 60 s before being immersed in deionized water overnight to allow for precipitation.

### 2.3. Membrane Characterization

#### 2.3.1. Scanning Electron Microscopy (SEM) Analysis

The cross sections, top and bottom surfaces of the membrane were characterized by using a HITACHI Tabletop SEM (TM3000, Tokyo, Japan). The membranes samples were dried at room temperature and were cryogenically fractured using liquid nitrogen to observe the cross-sectional image of samples. The membrane surface was coated under vacuum condition with a thin layer of gold (80%)/palladium (20%) to avoid electrostatic charging.

#### 2.3.2. Atomic Force Microscope (AFM) Analysis

The surface roughness of the membrane was investigated using AFM (Park System XE100, Suwon, Korea). The membrane mounted on a glass slide was scanned with a laser beam reflected by the cantilever. AFM was performed over 5 µm × 5 µm of the scanning area with a scanning rate of 0.25 Hz under tapping mode.

#### 2.3.3. Viscosity Analysis

The viscosity of the dope solution with various ZnO-NPs loading was measured using a Brookfield digital Rheometer (Model DV-III, Massachusetts, USA). The viscosity value of the dope solution was obtained at a temperature of 25 ± 2 °C and a shear rate of 10 s^−1^. The average viscosities of seven measurements were recorded.

#### 2.3.4. Porosity and Pore Size Determination

The porosity of the membrane was determined through its dry-wet weight. The membrane was immersed in water for 24 h. After that, the weight of the wet membrane was measured after wiping off excess water with filter paper. Then, the wet membranes were dried in an oven for 10 h at 25 °C and the weight of the dried membrane was measured. The porosity was calculated using the following equation [28]:

(1) $$\varepsilon \left(\%\right)=\frac{\left(w_{w}-w_{d}\right)/\rho_{w}}{\left(w_{w}-w_{d}\right)/\rho_{w}+w_{d}/\rho_{p}}\times 100$$

where ε is the membrane porosity, $w_{w}$ is the wet membrane weight (g), $w_{d}$ is the dry membrane weight (g), $\rho_{w}$ is the pure water density while $\rho_{p}$ is the polymer density.

The mean pore radius size ($r_{m}$) was calculated based on the pure water flux and porosity data obtained previously using the Guerout–Elford–Ferry equation as follows [29,30]:

(2) $$r_{m}=\sqrt{\frac{\left(2.9-1.75\varepsilon \right)8\eta lJ_{WF}}{\varepsilon \times A\times ∆P}}$$

where η is the viscosity of water, l is membrane thickness (m), while $J_{WF}$ is the pure water flux (g/m^2^·s), A is area of the membrane (m^2^), and ∆P is the operating pressure. Membrane thicknesses were measured using the Mitutoyo caliper ±2 μm.

#### 2.3.5. Contact Angle Measurements

The surface hydrophilicity of the membrane was characterized using the contact angle goniometer (Ramé-hart 200 Series, Ramé-hart, Succasunna, NJ, USA) at room temperature using deionized water. The hanging drop method was used to measure the contact angle on the membranes surfaces. A drop of water (1 μL) was deposited on the surface of the membrane using a motor. In order to establish the balances of forces involved, the contact angle reading was obtained after 10 s of the water droplet. To reduce experimental error, a series of seven measurements for each sample was taken and their mean values were calculated.

### 2.4. Membrane Performance Evaluation

#### 2.4.1. HA Feed Solution Preparation and Characterization

HA solution was used as a foulant in this study. HA solution was prepared by dispersing 0.1 g of HA in 2 L of deionized water. To aid the HA dispersion in water, the solution was sonicated for 1 h. The solution was stirred vigorously before used for membrane rejection test. The concentration of HA was measured using a UV spectrophotometer Pharo 300 (Merck, MA, USA) at a wavelength of 254 nm.

#### 2.4.2. Membrane Permeation Test and Fouling Analysis for Membrane

The membrane permeation test was carried out using a dead-end filtration unit. The membrane sample was immersed in deionized water for 1 day before being used in the membrane testing rig. Initially, the membrane sample was compressed for 30 min at a pressure of 10 bar. Then, the pure water flux measurement was performed at a pressure of 9 bar for 1 h. The initial pure water flux was calculated as follows:

(3) $$J_{WF}=\frac{m}{A_{m}t}$$

where m is the mass of permeate (g), $A_{m}$ is the effective filtration area (m^2^), and t is the measurement time (s).

The filtration was continued by replacing the pure water with the prepared HA at a pressure of 9 bar. The HA concentration before filtration was measured as mentioned in Section 2.4.1. The filtration of HA was performed for 1 h and the concentration of the permeate was measured at the end of the experiment. The flux was calculated using Equation (3). The anti-fouling capability of the membrane was evaluated through the relative flux reduction (*RFR*) which was calculated using Equation (4).

(4) $$RFR\left(\%\right)=\left(1-\frac{J_{HA}}{J_{WF}}\right)\times 100$$

where $J_{HA}$ is the permeate flux (g/m^2^·s) of tested solution (HA solution) and $J_{WF}$ is the initial water flux.

The membrane was washed using deionized water for 15 min and then the filtration was continued. The pure water flux measurement was performed again at a pressure of 9 bar for 1 h. The pure water flux after flux was evaluated using Equation (3). This was intended to evaluate the flux recovery ratio (*FRR*) of the membrane using Equation (5):

(5) $$FRR\left(\%\right)=\frac{J_{WF2}}{J_{WF}}\times 100$$

where *J_WF_*_2_ is the pure water flux (g/m^2^·s) after the washing step.

The fouling resistance of the membranes was calculated using Darcy’s law as shown in Equation (6).

(6) $$J_{WF}=\frac{TMP}{\mu \Sigma R}=\frac{TMP}{\mu R_{t}}$$

where TMP is the transmembrane pressure (Pa), μ is the permeate viscosity (Pa·s) and ΣR is similar to $R_{t}$ which is the total resistance (m^−1^).

The total resistance includes the fouling resistance ($R_{f}$) and intrinsic membrane resistance ($R_{m}$). For $R_{m}$, it was calculated using the Equation (8).

(7) $$R_{t}=R_{m}+R_{f}$$

(8) $$R_{m}=\frac{TMP}{\mu J_{WF}}$$

The $R_{f}$ consist of the fouling and concentration polarization effect. These two effects are considered as one factor due to the difficulty to differentiate them. $R_{f}$ is assumed to be the summation of the reversible ($R_{r}$) and irreversible ($R_{ir}$) fouling resistance. The concept is outlined in Equations (9)–(12).

(9) $$R_{f}=R_{r}+R_{ir}$$

(10) $$R_{f}=\frac{TMP}{\mu J_{HA}}-R_{m}$$

(11) $$R_{ir}=\frac{TMP}{\mu J_{WF2}}-R_{m}$$

(12) $$R_{r}=R_{f}-R_{ir}$$

## 3. Results and Discussion

### 3.1. Morphological Variation of Membrane

Figure S1 shows that PZ1 to PZ5 membranes have a finger-like macro void at the substructure, followed by the sponge-like bottom. The difference between the membranes is not obvious and all of them seem to have similar morphology. When the addition of ZnO-NPs exceeded 1.0 wt%, obvious cluster/agglomeration of ZnO was visible on the top layer and in the cross-section of PZ4 and PZ5 membranes. As reported by Balta et al. and Dipheko et al. and, it is common for the clustering of NPs to occur when the addition of NPs is increased beyond a certain limit [10,21], but serious agglomeration may lead to pore plugging.

### 3.2. Pore Size and Porosity

Based on Table 2, the porosity increases from PZ1 to PZ3 (1.0 wt% of ZnO), but further addition of ZnO in the membrane led to a decrease in porosity. The presence of ZnO-NP induces both the viscosity effect and hydrophilicity effect [31]. ZnO has a high affinity for water and can easily draw water into the casting suspension. Hence, it can increase the exchange rate between non-solvent (water) and solvent. However, the viscosity also keeps increasing as the addition of ZnO-NPs increase. The increase in viscosity of the PES/ZnO membrane when the amount of ZnO exceeds a certain level was also observed by researchers such as Nasrollahi et al. and Shen et al. [8,31]. Highly hydrophilic ZnO favors the formation of macro voids and as a consequence enhances the porosity. However, as the viscosity significantly increases from PZ3 to PZ5, membranes (PZ4 and PZ5) become less porous due to kinetic hindrance in the exchange of NMP/acetone and water. Initially, the hydrophilic effect of ZnO was dominant but the effect of viscosity started to take place after the ZnO concentration in the membrane exceeds 1.0 wt%. Furthermore, the decline in pore size could be due to clogging of pores caused by the ZnO agglomeration as mentioned earlier. Studies also have confirmed that higher loading of ZnO could lead to pore blockage in the membrane [20,32].

### 3.3. Contact Angle and Surface Roughness

**Figure 1:**
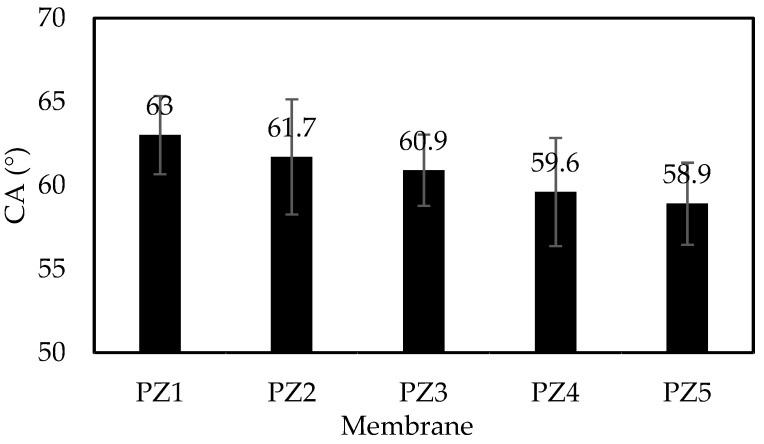
The contact angle of membrane PZ1 to PZ5.

**Figure 2:**
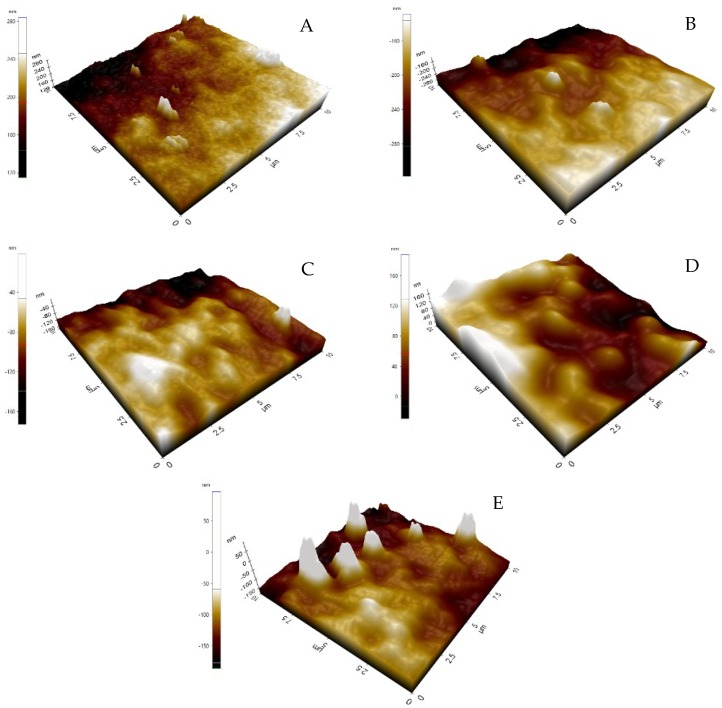
The atomic force microscope (AFM) image of surface roughness of membrane (**A**) PZ1, (**B**) PZ2, (**C**) PZ3, (**D**) PZ4 and (**E**) PZ5.

Figure 1 shows that membranes with ZnO-NPs loading (PZ2 to PZ5) have a lower contact angle value compared to the pristine membrane PZ1. A consistent decrease in the contact angle with the addition of ZnO-NPs proved that the addition of hydrophilic ZnO-NPs on the surface of the PES membrane improves the hydrophilicity of the membrane. The improved wettability of the membrane may increase the water flux, but, other factors such as pore size also have significant influence on the water permeation. Surface roughness analysis from AFM (Figure 2) was obtained to further investigate the characteristics of the surface of the membrane.

Table 3 shows that the surface roughness *R_a_* of the membrane increases with ZnO addition but the increment was not obvious and PZ1 to PZ3 membranes seem to have nearly the same roughness. For PZ4 and PZ5 membranes, the agglomeration slightly increases the roughness. Overall, all the membranes have very little difference in roughness due to the incorporation of acetone. The acetone modified membrane gives a smoother membrane due to the volatility of acetone. Partial evaporation of volatile acetone causes the polymer concentration to significantly increase at the top layer of the membrane forming a nearly defect-free, tight skin layer [33]. Ong et al. stated that the surface roughness of membranes prepared from mixed solvent (less volatile and more volatile solvent) is generally smoother than the membranes formed from pure NMP only at moderate evaporation temperature [34].

### 3.4. Performance Evaluation of the Membrane

Initially the pure water flux (PWF) of the membrane increases, but it gradually decreases after reaching a peak. The two major factors that affect the PWF are porosity and hydrophilicity. The combined effect of increased hydrophilicity and porous membrane structure leads to an increase in flux. The drastic drop of flux from PZ3 to PZ5 is as a result of ZnO-NPs accumulation and decreased porosity. Dispersion of ZnO-NPs on the membrane offers a larger surface area of NPs which leads to adsorption of more water molecules on the surface of the membrane. In contrast to that, a large amount of NPs which has a tendency for aggregation cause the effective surface of NPs to reduce and therefore hydroxyl groups on the surface of membranes to decline [35]. So, the agglomeration of ZnO which is evident in PZ4 and PZ5 membrane contributes to the decline in water flux for the respective membranes.

**Figure 3:**
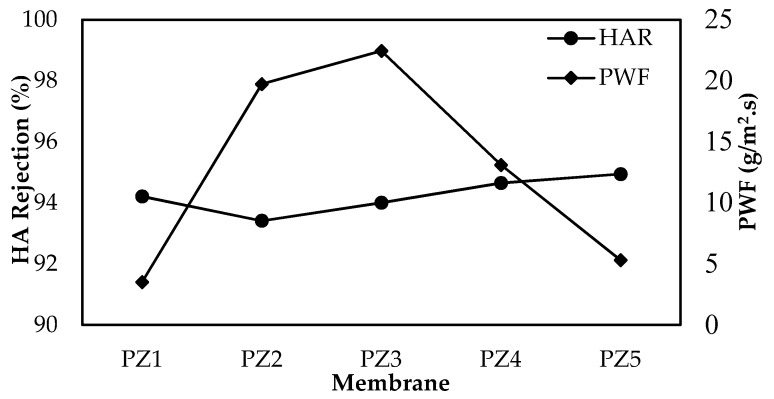
The trade-off graph of pure water flux (PWF) and rejection of humic acid (HA) of membrane PZ1 to PZ5.

Referring to Figure 3, the rejection of HA decreased from PZ1 to PZ2 but the rejection slowly improved. The decrease in pore size and tight skin layer could improve the rejection of HA. Aside from that, increasing the hydrophilicity of the membrane decreases the adsorption of hydrophobic HA onto the membrane and hence the rejection potential increases. A lower concentration of HA in permeate is maybe due to slower diffusion of HA solute through membrane PZ2 to PZ5 than the neat membrane (PZ1). Balta et al. suggested that an effective way to improve the rejection of hydrophobic organic matters is to avoid hydrophobic interaction at the interface of the solute–membrane surface [10].

### 3.5. Fouling Evaluation of Membrane

A higher value of FRR reflects a lower level of persistent HA adsorption onto the membrane surface. The lower the RFR value, the lower the fouling tendency of the membrane. Based on Table 4, the membrane modified with ZnO shows a lower FRR value compared to the unmodified. The FRR value of ZnO modified membranes improves as the addition of ZnO increase. Similarly, the RFR value of PZ1 is the lowest among all the membranes. The RFR value continuously increases up to 1.0 wt% of ZnO loading (PZ3), but the value decreases with further addition of NPs. It is interesting to note that the membrane (PZ1) modified with acetone-only has the highest FRR and lowest RFR value than the other ZnO–Acetone membranes, signifying the best antifouling ability. The large pore of PZ2 and PZ3 membrane could also contribute to the lower FRR value since HA molecule can strongly adsorb at inner pore cause pore narrowing [36].

**Figure 4:**
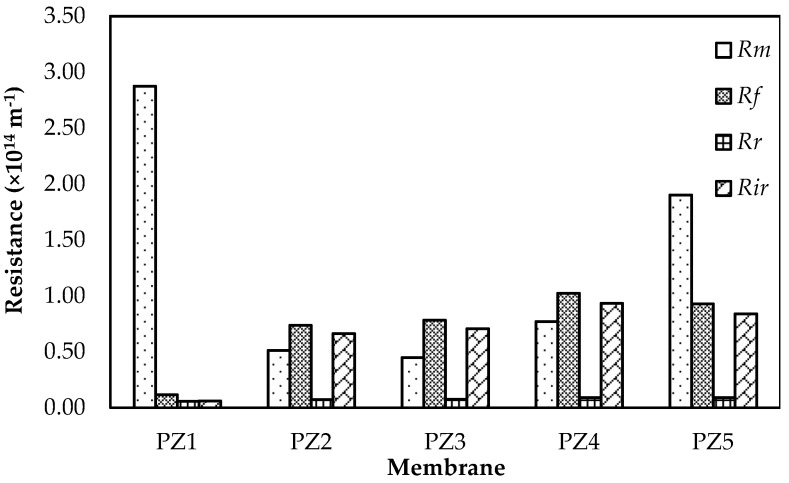
The membrane resistance *R_m_*, *R_f_*, *R_r_* and *R_ir_* of PZ1 to PZ5.

To enable a detailed discussion on fouling, the resistance of membranes such as *R_m_*, *R_f_*, *R_r_*, and *R_ir_* are evaluated and presented in Figure 4. As observed, the value of Rm decreases from PZ1 to PZ3 but increases from PZ3 to PZ5. Kinetic hindrance for the flow which is caused by the small and clogged pores as the ZnO addition increases provides more obstacles in the liquid flowing path causing the membrane resistance to increase. The reversible fouling resistance Rr of all the membrane is relatively similar. A very insignificant increase in roughness between the membrane did not give any impact to the reversible fouling. While the irreversible fouling has a noticeable increase from the neat membrane to PZ1, the membrane prepared with only acetone has a tight skin layer with little open pores. Although PZ1 is more hydrophobic than PZ2, the porosity and pore size played an important role in determining the severity of irreversible fouling to the membrane in this case. Katsoufidou et al. stated that accelerated irreversible fouling occurs due to internal pore adsorption [37]. The size exclusive effect and the surface gel layer effect of the tight pore skin layer of PZ1 might prevent thrusting of HA molecules causing inner pore adsorption. Lin et al. also mentioned that surface gel layer formation with very little inner pore adsorption is the primary form of fouling in tight pore membranes [38]. Addition of ZnO encourages the formation of more open pores which increases the surface area for adsorption of HA. Zhao et al. and Aryanti et al. have also shown that an open, large pore is more prone to fouling compared to small pores [39,40].

The ZnO-Acetone membranes show good performance in terms of flux and rejection of humic acid. But, the FRR value of these membranes is lower compared to the membranes prepared with only acetone. Acetone alone is capable of producing a membrane with high antifouling capability and the membrane has a lower level of irreversible and reversible fouling than the ZnO-Acetone membranes. Although the FRR value improves with 2.0 wt% loading of ZnO (PZ5) (69.3%), the value is still lower compared to the membrane prepared with only acetone by 28.55%.

Table 5 shows the comparison of this study with current similar studies. Other studies used different types of additives to enhance the properties of the membranes. In this study, we only used two different solvents to create a better membrane. The comparison is made to have a deeper knowledge of the influence of ZnO on the membrane characteristics and antifouling properties. The data presented in Table 5 clearly shows that the addition of ZnO nanoparticles influenced the membrane characteristics and performance regardless of the types of polymer used as membrane materials. Nevertheless, since there is a large variation in the conditions in which the studies were performed, it was difficult to make a general deduction.

## 4. Conclusions

In this study, the effect of ZnO in a mixed solvent (NMP and acetone) on the morphology and performance of membranes was studied. The agglomeration of ZnO at a higher concentration cannot be solved by even a dual-solvent system (NMP and acetone). The dispersion of ZnO in this dual solvent is questionable since the agglomeration was really visible in the SEM image at higher concentration. ZnO in the presence of acetone, is able to produce a membrane with high hydrophilicity and porosity. The flux was tremendously improved and rejection slightly improved in these membranes. However, the antifouling ability of the membrane prepared with only acetone was greater than the ZnO–acetone modified membranes. The acetone which creates a tight skin layer reduces fouling better than the hydrophilic ZnO. The potential of acetone as an antifouling agent was identified and further studies regarding its effect can be carried out by varying acetone concentration with a fixed amount of ZnO to obtain further knowledge on the antifouling capability of acetone in the presence of ZnO. Although the antifouling ability of ZnO/acetone is moderate, it is still plausible to obtain good membranes using these two materials since acetone shows a remarkable fouling resistance.

## Figures and Tables

**Table 1:** The recipe of the cast solution.

Membrane	PES Weight Percent (wt%)	NMP: Acetone Ratio of Solvent	ZnO Weight Percent (wt%)
PZ1	18	1:0.05	0
PZ2	18	1:0.05	0.5
PZ3	18	1:0.05	1.0
PZ4	18	1:0.05	1.5
PZ5	18	1:0.05	2.0

**Table 2:** The porosity, mean pore radius, and viscosity of PZ1 to PZ5.

Membrane	Porosity (%)	Mean Pore Size (nm)	Viscosity (cP)
PZ1	37.29 ± 2.86	6.80 ± 1.45	920 ± 10
PZ2	42.87 ± 1.24	14.01 ± 3.45	950 ± 5
PZ3	47.34 ± 3.24	13.96 ± 5.78	990 ± 10
PZ4	43.73 ± 6.32	11.26 ± 3.42	1160 ± 15
PZ5	41.87 ± 2.34	7.38 ± 2.52	1290 ± 10

**Table 3:** The statistical analysis of membrane roughness for membrane PZ1 to PZ5.

Membrane	*R_a_* (nm)	*R_q_* (nm)	*R_z_* (nm)
PZ1	23.73 ± 3.25	29.36 ± 3.43	165.37 ± 4.47
PZ2	24.28 ± 6.15	29.98 ± 8.53	184.88 ± 4.32
PZ3	24.74 ± 6.75	30.63 ± 7.21	182.55 ± 4.31
PZ4	25.96 ± 3.22	36.98 ± 2.64	210.17 ± 6.53
PZ5	25.78 ± 5.20	31.77 ± 2.98	278.09 ± 2.41

Average roughness (*R_a_*), root mean square of Z data (*R_q_*) and mean difference between the highest peaks and the lowest valleys across the scanned area (*R_z_*).

**Table 4:** The numerical analysis of initial water flux *J_WF_*, humic acid (HA) filtration *J_HA_*, second water flux *J_WF_*_2_, flux recovery ratio (FRR) and relative flux reduction (RFR) for membrane PZ1 to PZ5.

Membrane	*J_WF_* (g/m^2^·s)	*J_HA_* (g/m^2^·s)	*J_WF_*_2_ (g/m^2^·s)	*FRR* (%)	*RFR* (%)
PZ1	3.51	3.37	3.44	97.93	3.94
PZ2	19.72	8.08	8.59	43.55	59.03
PZ3	22.44	8.19	8.72	38.89	63.52
PZ4	13.09	5.62	5.92	45.24	57.06
PZ5	5.30	3.56	3.68	69.38	32.83

**Table 5:** Comparison between this study and similar studies.

Casting Conditions and Membrane Characteristics	Nasrollahi et al. (2018) [8]	Zinadini et al. (2017) [41]	Chung et al. (2017) [42]	Rabiee et al. (2015) [32]	This Work
Polymer	PES	PES	PSF	Polyvinyl Chloride (PVC)	PES
Polymer dosage (wt%)	18	20	20	15	18
Solvent	DMAc	DMAc	NMP	NMP	NMP and Acetone (0.05 mass ratio of NMP to acetone in a mixed solvent)
Additive and dosage	PVP (2 wt%) and Copper oxide (CuO)	PVP (1 wt%) and MWCNTs	GO	Polyethylene glycol (PEG 6 kDa); 4 wt%	-
ZnO dosage (wt%)	0.1, 0.2, 0.5 and 1.0 ^a^	0.1, 0.5 and 1.0 ^b^	0.1, 0.3 and 0.6 ^c^	0.3, 1.0, 2.0, 3.0 and 4.0	0.5, 0.1, 1.5 and 2.0
Contact angle (°)	66.5 (0.2 wt% CuO/ZnO)	57.2 (0.5 wt% ZnO/MWCNTs)	40 (0.6 wt% ZnO/GO)	54.5 (3 wt% ZnO)	60.9 (0.1 wt% ZnO)
Foulant	Bovine Serum Albumin (BSA), (500 mg/L)	Powder milk (8000 ppm)	Humic acid (10 ppm)	BSA (500 ppm)	Humic acid (50 mg/L)
Rejection (%)	99 (0.2 wt% CuO-ZnO)	95 (0.5 wt% ZnO/MWCNTs) ^d^	99 (0.6 wt% ZnO/GO)	97.5 (3 wt% ZnO)	94 (0.1 wt% ZnO)
Pure water flux	679 kg/m^2^·h (0.2 wt% CuO-ZnO)	16.7 kg/m^2^·h (0.5 wt% ZnO/MWCNTs)	5.11 kg/m^2^·h·bar (0.6 wt% ZnO/GO)	401.9 kg/m^2^·h (3 wt% ZnO)	80 kg/m^2^·h (0.1 wt% ZnO)
Flux recovery ratio	50.1 (0.2 wt% CuO-ZnO)	88.6 (0.5 wt% ZnO/MWCNTs)	99 (0.6 wt% ZnO/GO)	91.8 (3 wt% ZnO)	38.89 (0.1 wt% ZnO)

^a^ The percentage represents ZnO/CuO nanocomposite. ^b^ The percentage represents ZnO coated multiwalled carbon nanotube nanocomposite. ^c^ The percentage represents ZnO-GO nanohybrid which produced via sol-gel method by incorporating 20 wt% of ZnO onto the GO nanosheets support. ^d^ This is the rejection percentage of Direct Red 16 dye at pH = 6 and 30 ppm concentration.

## Supplementary Materials

The following are available online at http://www.mdpi.com/2077-0375/8/4/131/s1, Figure S1: SEM micrographs of PZ1, PZ2, PZ3, PZ4, and PZ5 membrane (i). cross-section at 1200× magnification and (ii). Top surface at 2000× magnification.
