# Supplementary material for: Antifouling Properties of PES Membranes by Blending with ZnO Nanoparticles and NMP–Acetone Mixture as Solvent
Source: Membranes (Basel). 2018 Dec 14;8(4):131. doi: 10.3390/membranes8040131 (PMC6316317; doi:10.3390/membranes8040131)
Supplement: Supplementary file 1 [file membranes-08-00131-s001.pdf]

Supplementary Materials: Antifouling Properties of PES Membranes by Blending with ZnO Nanoparticles and NMP–Acetone Mixture as Solvent

Abdul Latif Ahmad [1,*], Jayasree Sugumaran [1] and Noor Fazliani Shoparwe [2]

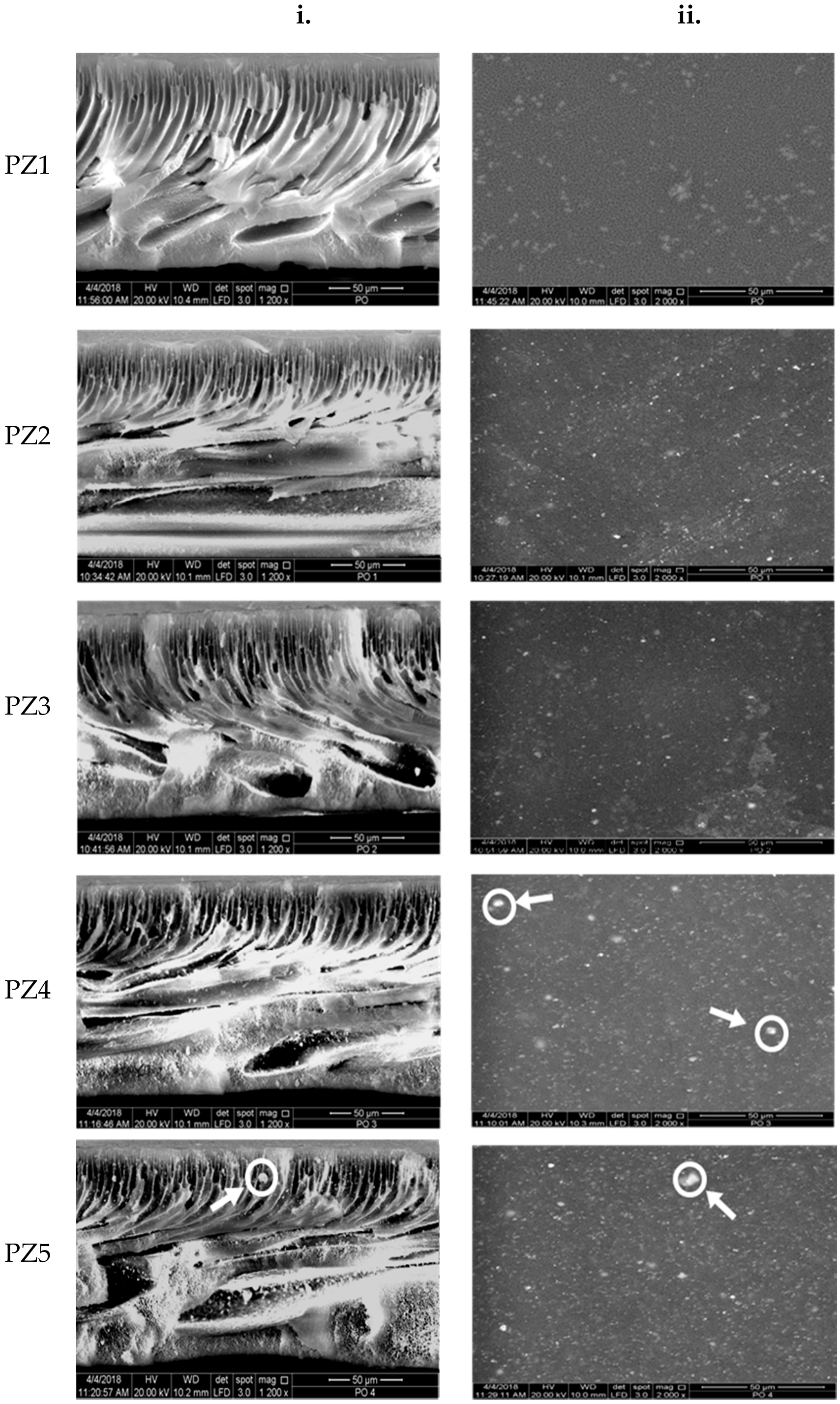

Figure S1. SEM micrographs of PZ1, PZ2, PZ3, PZ4 and PZ5 membrane (**i**) cross-section at 1200× magnification and (**ii**) Top surface at 2000× magnification.
